# Coronary microvascular dysfunction as an initial clue to the diagnosis of light-chain amyloidosis: a case report

**DOI:** 10.1093/ehjcr/ytaf631

**Published:** 2025-12-02

**Authors:** Ryo Bando, Shusuke Yagi, Muneyuki Kadota, Takayuki Ise, Masataka Sata

**Affiliations:** Department of Cardiovascular Medicine, Tokushima University Graduate School of Biomedical Sciences, 3-18-15, Kuramoto-chou, Tokushima 770-8503, Japan; Department of Cardiovascular Medicine, Tokushima University Graduate School of Biomedical Sciences, 3-18-15, Kuramoto-chou, Tokushima 770-8503, Japan; Department of Cardiovascular Medicine, Tokushima University Graduate School of Biomedical Sciences, 3-18-15, Kuramoto-chou, Tokushima 770-8503, Japan; Department of Cardiovascular Medicine, Tokushima University Graduate School of Biomedical Sciences, 3-18-15, Kuramoto-chou, Tokushima 770-8503, Japan; Department of Cardiovascular Medicine, Tokushima University Graduate School of Biomedical Sciences, 3-18-15, Kuramoto-chou, Tokushima 770-8503, Japan

**Keywords:** Coronary microvascular dysfunction, AL amyloidosis, Index of microvascular resistance, Case report

## Abstract

**Background:**

Coronary microvascular dysfunction (CMD) is a recognized cause of ischaemia in patients without obstructive coronary artery disease; however, it may also present as a clinical manifestation of light-chain (AL) amyloidosis.

**Case summary:**

A 67-year-old woman presented with exertional chest discomfort. Coronary computed tomography suggested coronary artery stenosis, and the clinical presentation initially indicated angina pectoris. However, coronary angiography revealed no obstructive lesions. An invasive physiological assessment showed a severely reduced coronary flow reserve (1.0) and a markedly elevated index of microvascular resistance (IMR, 77), findings that were consistent with CMD. The initiation of a beta-blocker worsened these symptoms and increased the B-type natriuretic peptide level. In further evaluations, a monoclonal IgG-κ protein was identified, technetium-99m pyrophosphate scintigraphy was negative, and bone marrow biopsy showed amyloid deposits. Endomyocardial biopsy confirmed AL-type cardiac amyloidosis. The patient was referred to the Department of Hematology, and chemotherapy was initiated.

**Discussion:**

The present case suggests that CMD with a markedly elevated IMR is an early sign of AL amyloidosis. An awareness of this relationship may help avoid inappropriate therapies, such as beta-blockers, and enable an earlier diagnosis and treatment

Learning points

- Coronary microvascular dysfunction (CMD) with a markedly elevated index of microvascular resistance may be the first manifestation of AL cardiac amyloidosis.
- In patients with CMD and no epicardial coronary stenosis, infiltrative cardiomyopathy needs to be considered early in the diagnostic work-up.

## Introduction

Coronary microvascular dysfunction (CMD) is a recognized cause of ischaemic symptoms in patients without obstructive coronary artery disease.^1–3^ Recent studies suggest that CMD also reflects myocardial involvement in infiltrative diseases, particularly amyloidosis.^4–6^ In light-chain (AL) amyloidosis, amyloid deposition frequently occurs around small intramyocardial vessels,^7,8^ resulting in increased microvascular resistance and ischaemia. We herein present a case of AL amyloidosis in which CMD was the first diagnostic clue, leading to further investigations and an early diagnosis.

## Summary figure

**Table:** 

Time	Event/findings
**Several months before the first visit**	Chest discomfort on mild exertion
**First visit**	Progressive chest discomfort, even with minimal activity
**Initial work-up**	Coronary computed tomography: possible left circumflex artery stenosis Coronary angiography: no significant epicardial stenosis Coronary microvascular function test: CFR, 1.0; IMR, 77
**Treatment**	Beta-blocker (bisoprolol, 1.25 mg/day) initiated
**After initiation**	Worsening symptoms → beta-blocker discontinued Calcium channel blocker + loop diuretic started → symptoms improved
**Further evaluation**	Serum/urine immunofixation: a monoclonal IgG-κ protein and Bence–Jones protein detected technetium-99m pyrophosphate (99mTc-PYP) scintigraphy: negative Random skin biopsy: negative Bone marrow biopsy: plasma cells < 10% (multiple myeloma excluded)
**Final diagnosis**	Right ventricular endomyocardial biopsy: AL cardiac amyloidosis → referred to haematology
**Treatment**	Daratumumab + cyclophosphamide + bortezomib + dexamethasone (the DaraCyBorD regimen) initiated
**Follow-up**	No recurrence of chest symptoms

## Case report

**Figure 1:**
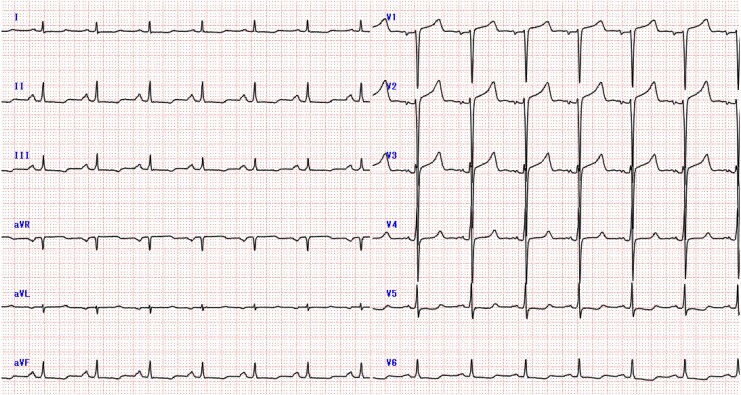
Electrocardiography showed a low voltage, poor R wave progression, and mild ST depression in leads I, aVL, and V5–6.

**Figure 2:**
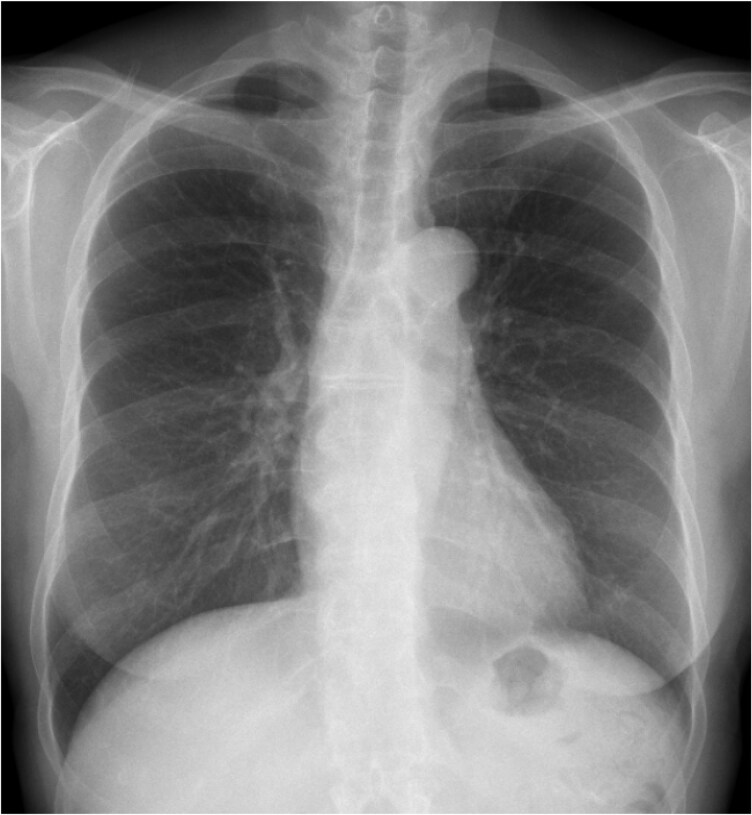
Chest radiography showed no signs of pulmonary congestion or enlargement of the heart shadow.

**Figure 3:**
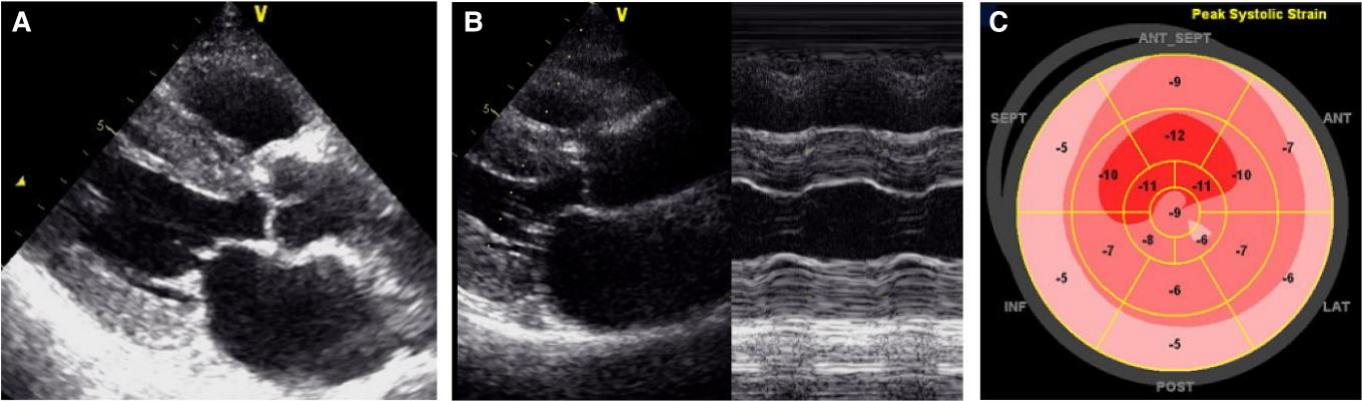
Echocardiographic findings demonstrating diffuse thickening of the left ventricular wall. Measurements: interventricular septum thickness, 12.3 mm; posterior wall thickness, 12.4 mm; left ventricular end-diastolic diameter, 37 mm; left ventricular end-systolic diameter, 27 mm; and left ventricular ejection fraction, 57%. (*A*) Parasternal long-axis view. (*B*) M-mode imaging. (*C*) Left ventricular longitudinal strain.

A 67-year-old woman with a history of hypothyroidism presented to our hospital with chest discomfort during mild exertion. In the preceding months, her symptoms had progressively worsened, and she experienced discomfort even with minimal activity, such as walking 20–30 m or climbing stairs. She had no history of smoking, alcohol use, or allergies and was taking levothyroxine at a dose of 25 μg/day. Her blood pressure was 113/75 mmHg, and her pulse rate was 78 beats per minute. No cardiac murmurs were noted, and a lung examination revealed no rales. Initial blood tests revealed mildly elevated cardiac enzymes, including creatine kinase (CK) at 269 U/L, CK-MB at 5.5 ng/mL, and high-sensitivity troponin T at 0.026 ng/mL. Brain natriuretic peptide (BNP) was also elevated, measuring 465.3 pg/mL. Additional laboratory findings revealed a LDL cholesterol level of 100 mg/dL and a haemoglobin A1c of 5.8%. Electrocardiography showed a low voltage, poor R wave progression, and mild ST depression in leads I, aVL, and V5–6 (*Figure 1*). Chest radiography revealed no cardiomegaly or pulmonary congestion (*Figure 2*). Transthoracic echocardiography detected concentric left ventricular hypertrophy (LVH) with preserved systolic function (interventricular septum, 12.3 mm; posterior wall, 12.4 mm; left ventricular end-diastolic diameter, 37 mm; left ventricular end-systolic diameter, 27 mm; and left ventricular ejection fraction, 57%) (*Figure 3*). There was no evidence of left ventricular outflow tract obstruction or aortic stenosis (AS). The right ventricle demonstrated preserved systolic function (tricuspid annular plane systolic excursion, 17 mm) without hypertrophy. No clear apical sparing pattern was observed.

Coronary computed tomography suggested stenosis of the left circumflex artery. Based on the patient’s symptoms, angina pectoris was suspected, and coronary angiography was performed. However, no significant stenosis of the epicardial coronary arteries was observed. A physiological assessment showed a severely reduced coronary flow reserve (CFR, 1.0) and a markedly elevated index of microvascular resistance (IMR, 77), indicating CMD.

**Figure 4:**
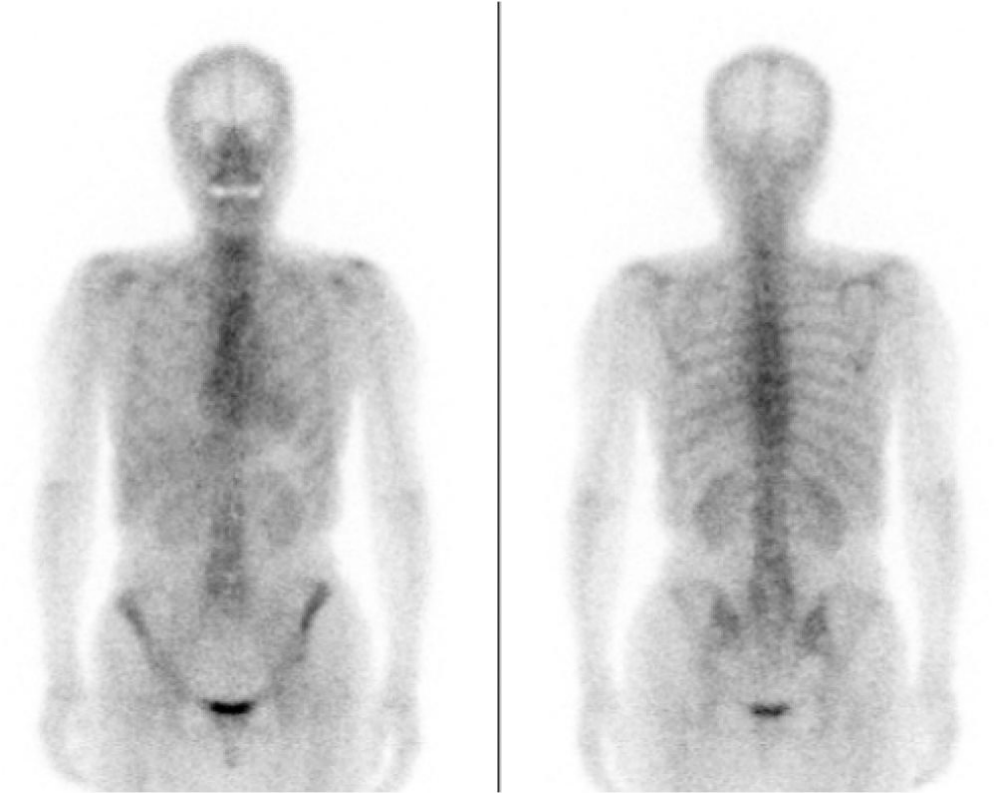
Technetium-99m pyrophosphate scintigraphy demonstrated mild myocardial uptake (heart-to-contralateral ratio = 1.26, Grade 1), less intense than rib uptake, consistent with a negative finding for cardiac amyloidosis. H/CL, heart-to-contralateral.

**Figure 5:**
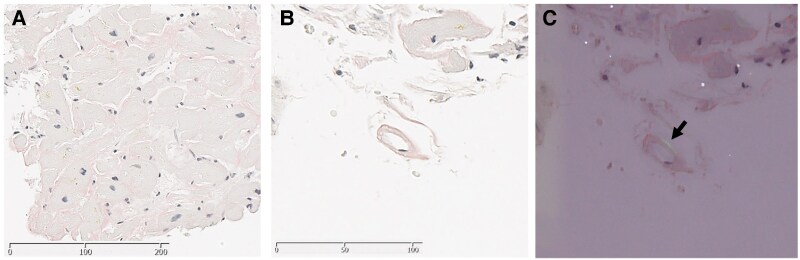
Morphological patterns of amyloid deposition in endomyocardial biopsy specimens. (*A*) Congo red staining (original magnification ×400) showed amyloid protein deposition in the interstitial tissue. (*B*) Congo red staining highlighting the microvasculature (original magnification ×800). (*C*) Congo red staining viewed under polarized light microscopy. The arrow indicates amyloid protein deposition in the vascular adventitia.

A beta-blocker (bisoprolol, 1.25 mg/day) was initiated; however, symptoms worsened. Brain natriuretic peptide increased to 795 pg/mL, and chest radiography showed pulmonary congestion. After the discontinuation of the beta-blocker and the initiation of a dihydropyridine calcium channel blocker along with a loop diuretic, symptoms improved. Due to the atypical clinical course, imaging findings, and elevated IMR, a type of cardiomyopathy, particularly cardiac amyloidosis, was suspected. Cardiac magnetic resonance imaging (CMR) was not performed due to the patient’s claustrophobia. Serum and urine immunofixation revealed a monoclonal IgG-κ protein and Bence–Jones protein. Technetium-99m pyrophosphate scintigraphy was negative (*Figure 4*). Bone marrow biopsy showed <10% plasma cells, ruling out multiple myeloma, and a diagnosis of AL amyloidosis was made. However, the sample was insufficient for definitive subtyping. Skin biopsy performed at a random site was negative. Right ventricular endomyocardial biopsy confirmed the diagnosis of AL cardiac amyloidosis (*Figure 5*). The patient was referred to the Department of Hematology, where chemotherapy with the combination of daratumumab, cyclophosphamide, bortezomib, and dexamethasone (the DaraCyBorD regimen) was initiated. Following treatment, the M-protein became undetectable. She remains under outpatient follow-up, with no recurrence of chest symptoms to date.

## Discussion

Coronary microvascular dysfunction is increasingly recognized as a cause of myocardial ischaemia in the absence of obstructive coronary artery disease.^1–3^ It is typically diagnosed using physiological indices, most notably CFR and IMR, with thresholds of CFR < 2.0 and IMR ≥ 25 commonly being used.^9–11^ Recent studies have reported associations between CMD and cardiomyopathies associated with LVH, such as hypertensive heart disease, hypertrophic cardiomyopathy, AS, Fabry disease, and cardiac amyloidosis.^4–6^ In the present patient, echocardiography demonstrated LVH; however, there was no history of significant hypertension, no evidence of left ventricular outflow tract obstruction or valvular stenosis on echocardiography, and no extracardiac manifestations suggestive of Fabry disease. In addition, there was no family history suggestive of Fabry disease. Furthermore, the discordance between LVH and low QRS voltage on electrocardiography supported the diagnosis of cardiac amyloidosis. Although CMR was not performed, it remains an important modality for evaluating the aetiology of LVH and should be conducted when feasible.

The patient exhibited a markedly elevated IMR of 77, indicating significant microvascular dysfunction. Coronary microvascular dysfunction has been reported in both AL and transthyretin (ATTR) amyloidosis.^4–6^ In ATTR amyloidosis, CMD is thought to result mainly from interstitial amyloid deposition, whereas in AL amyloidosis, the predominant perivascular amyloid accumulation may cause more direct microvascular injury.^7,8^ This difference in amyloid distribution could explain the more severe impairment of the coronary microvasculature observed in AL amyloidosis. Suiko *et al.*^12^ reported an average IMR of 35.4 ± 3.0 in a cohort of 30 patients with cardiac ATTR amyloidosis, which was considerably lower than that observed in our patient. Furthermore, Choi *et al.*^13^ demonstrated that an IMR > 40 in patients with AL amyloidosis was associated with worse survival outcomes, underscoring the prognostic value of this parameter. Therefore, when CMD is identified, cardiac amyloidosis, including both AL and ATTR types, should be considered in the differential diagnosis. Among these, a markedly elevated IMR may suggest AL amyloidosis and could reflect advanced microvascular involvement and an unfavourable prognosis.

The pharmacological management of CMD typically includes beta-blockers to reduce the myocardial oxygen demand.^14^ However, in cardiac amyloidosis, particularly the AL type, beta-blockers may exacerbate symptoms due to autonomic dysfunction and a low-output state.^15^ In the present case, the initiation of bisoprolol led to worsening symptoms and elevated BNP levels. These adverse effects might have been avoided if amyloidosis had been suspected earlier, highlighting the importance of considering alternative diagnoses in CMD with atypical features. Beyond absolute CFR and IMR values, the CMD classification using integrated physiological parameters, as proposed by Verma *et al*.,^6^ may facilitate the recognition of underlying pathologies, including infiltrative cardiomyopathies. This approach may help avoid inappropriate treatments, such as premature beta-blocker use, and promote earlier diagnostic interventions in complex cases, such as ours.

The present case demonstrates that CMD, particularly when accompanied by markedly elevated IMR, may serve as an early indicator of infiltrative cardiomyopathies, such as AL amyloidosis. Although CMD as an initial manifestation of cardiac amyloidosis has infrequently been reported, its growing recognition supports incorporating CMD assessment into early diagnostic workflows. Integrating invasive physiological data with imaging and laboratory findings may enhance diagnostic accuracy.

In conclusion, the evaluation of CMD not only facilitates the diagnosis of microvascular angina but may also uncover underlying systemic disorders such as amyloidosis. In patients presenting with exertional chest discomfort, preserved epicardial coronary arteries, and a disproportionately elevated IMR, infiltrative cardiomyopathies, particularly AL amyloidosis, need to be considered. Coronary microvascular dysfunction may represent the earliest and, in some cases, the sole cardiac manifestation of AL amyloidosis. Early recognition is essential, as timely diagnosis and initiation of appropriate therapy can improve clinical outcomes. This case highlights the importance of considering cardiac amyloidosis as a potential underlying cause of chest symptoms initially attributed to CMD.

## Data Availability

The data underlying this article will be shared on reasonable request to the corresponding author.

## Lead author biography

Ryo Bando is an interventional cardiologist. He graduated from Tokushima University and received his M.D. in 2016. Since 2022, he has received cardiology training in the Department of Cardiovascular Medicine, Tokushima University Hospital, and serves as a member of the medical staff.
